# Multisystem reactions during egg oral food challenges may be associated with less severe reactions on initial presentation

**DOI:** 10.1186/s13223-016-0125-1

**Published:** 2016-04-28

**Authors:** Girish Vitalpur, Ann Esquivel, Kirsten M. Kloepfer, James E. Slaven, Frederick E. Leickly

**Affiliations:** Department of Pediatrics, Indiana University School of Medicine, 705 Riley Hospital Drive, ROC 4270, Indianapolis, IN 46202 USA; Department of Pediatrics, University of Wisconsin-Madison, Madison, USA; Department of Biostatistics and Public Health, Indiana University School of Medicine, Indianapolis, USA

**Keywords:** Egg allergy, Extensively heated (EH) egg allergy, Oral food challenge (OFC), Reaction severity, Type of reaction

## Abstract

In this study, we assessed whether multisystem reactions to egg and extensively-heated (EH) egg during OFCs were associated with a history of multisystem reactions. Records of children, who underwent OFC to egg or EH egg over a five-year period were reviewed. Of the 120 challenges, 26 (21.67 %) failed, with 38.4 % (10/26) having multisystem reactions. Of the 13 who had multisystem reactions on initial presentation, only two (15.4 %) had a similar OFC outcome. Eighty percent (8/10) of those who had a multisystem OFC reaction had a less severe initial presentation. Initial and OFC multisystem reactions were not associated with each other.

## Findings

Egg allergy affects 0.5–2.5 % of young children in the United States [1]. Up to 70 % percent of children with egg allergy tolerate extensively-heated (EH) egg [2, 3]. Patient history, skin prick tests (SPTs) and specific IgE (sIgE) values are used to determine whether an oral food challenge (OFC) should be performed. Systemic reactions to egg have been associated with poor prognosis of resolution of egg allergy [4]. In this study, we assessed whether multisystem reactions to egg on initial presentation were associated with similar OFC outcomes to egg or EH egg.

This project was approved by the University Human Subjects Committee. Records of children, who underwent OFC to egg or EH egg, from January 2008 to December 2012, were reviewed. OFCs were supervised by two allergists at an academic pediatric allergy clinic. OFCs were offered based on: history; SPT results; and/or sIgE values. Eligibility included lack of reaction to egg within the past 12 months. OFCs were offered to egg when egg white sIgE was ≤0.6 k U/L, i.e., when the negative predictive value was 90 % [5]. OFCs to EH egg were offered if the child had not been exposed to EH egg, regardless of SPT results or sIgE values. Pasteurized dried egg white powder (King Arthur, White River Junction, Vermont) was used for egg OFCs, with a cumulative dose of 12 g of egg protein, following an established protocol [6, 7]. For EH OFCs, families were instructed to prepare a muffin and a waffle at home, using published recipes. The muffin and waffle were given per established protocol [2]. All patients were observed for 1 h following the last dose. OFCs were stopped if there were symptoms suggestive of an allergic reaction, and treated accordingly.

Serum samples for IgE testing to egg white were analyzed by the Immunocap system (Thermo Fisher, Uppsala, Sweden). Skin prick tests were done per published standards [6].

Fisher’s exact tests were used on categorical outcomes, due to low cell counts. Mann–Whitney–Wilcoxon non-parametric tests were used on continuous outcomes, due to skewness of data. Statistical analysis was performed using SAS v9.3 (SAS Institute, Cary, NC). A p value of <0.05 was considered significant.

Ninety-five challenges were performed to egg, and 25 to EH egg, resulting in 120 challenges overall (Table 1). Of the 120 challenges, 26 (21.67 %) failed. Among egg OFCs, the failure rate was 23.16 % (22/95). Among EH egg OFCs, the failure rate was 16.00 % (4/25). Patient demographics and history are noted in Table 1.

**Table 1:** Clinical history and outcomes of patient population undergoing Egg and EH Egg OFCs

	Egg—Pass (n = 73)	Egg—Fail (n = 22)	p values (for egg)	EH egg—pass (n = 21)	EH egg—fail (n = 4)	p values (for EH egg)
Median age (years)	3.8 (1.0–16.8)	4.67 (1.23–13.3)	0.22	4.17 (2.3–10.0)	5.21 (3.9–8.7)	0.31
Gender			0.22			0.57
Male, no (%)	42 (44.2 %)	16 (16.8 %)		15 (60 %)	2 (8 %)	
Female, no (%)	31 (32.6 %)	6 (6.3 %)		6 (24 %)	2 (8 %)	
Ethnicity			0.97			1.00
White, no (%)	54 (56.8 %)	18 (18.9 %)		19 (76 %)	4 (16 %)	
Black, no (%)	6 (6.3 %)	2 (2.1 %)		2 (8 %)	0	
Other, no (%)	13 (13.7 %)	2 (2.1 %)		0	0	
Asthma, no (%)	27 (28.4 %)	9 (9.5 %)	0.80	8 (32 %)	4 (16 %)	0.04
AD, no (%)	52 (54.7 %)	14 (14.7 %)	0.60	11 (44 %)	4 (16 %)	0.12
Other food allergies, no (%)	58 (61.1 %)	16 (16.8 %)	0.56	14 (56 %)	4 (16 %)	0.29
Family history of atopy, no (%)	50 (52.6 %)	19 (20 %)	0.11	11 (44 %)	4 (16 %)	0.12
SPT^a,b^+, initial	87.6	88.2	1.00	96.1	100.0	1.00
SPT^a,b^+, at OFC	44.5	36.3	0.63	52.0	57.1	1.00
Initial egg white IgE, median,kU/L(range)	1.1 (0–10.9)	0.8 (0–19.7)	0.83	5.4 (0–69.6)	24.6 (6.4–100.0)	0.14
OFC Egg white IgE, median, kU/L (range)	0.0 (0–2.7)	0 (0–1.7)	0.05	2.3 (0–52.0)	52.4 (11.1–90.8)	0.02
Total	73 (76.8 %)	22 (23.2 %)		21 (84 %)	4 (18 %)	
Overall	95			25		

^a^% of overall population group

^b^SPTs were done with commercial extracts only. SPTs were not done with EH egg items

Of the 120 patients, the most common presenting reason to the allergy clinic was urticaria (26.7 %) (Table 2). If the initial reaction was multisystem in nature, the symptoms involved were hives and vomiting. None of the initial egg reactions involved an EH product.

**Table 2:** Initial presenting reaction vs reactions during OFCs for Egg and EH Egg

	Initial reaction			OFC reaction		
	EGG (n = 95)	EH EGG (n = 25)	P value	EGG (n = 22)	EH EGG (n = 4)	P value
Urticaria/angioedema	28 (29 %)	4 (16 %)	0.21	8 (36 %)	0	0.28
Multisystem reaction	9 (9 %)	4 (16 %)	0.47	6 (27 %)	4 (100 %)	0.01
GI reaction	9 (9 %)	3 (12 %)	0.71	6 (27 %)	0	0.54
Contact urticaria	9 (9 %)	1 (4 %)	0.69	0	0	1.00
Atopic dermatitis	3 (3 %)	8 (32 %)	0.0001	0	0	1.00
Oral itching or tingling^a^	0	0	1.00	1 (3.8 %)	0	1.00
Pruritus^a^	0	0	1.00	1 (3.8 %)	0	1.00
No history of exposure^b^	26 (27 %)	5 (20 %)	0.61	Na	Na	
Other reactions^c^	11 (12 %)	0 (0 %)	0.12	0	0	1.00

^a^Subjective complaints

^b^No history of exposure = no or unlikely exposure due to +SPT or egg IgE

^c^Other reactions = spitting out, or feeding refusal

Of the 26 overall OFC failures, the most common outcomes were multisystem reactions (38.4 %); (Table 2). Eighty percent (8/10) of the OFC-induced multisystem reactions involved urticaria and gastrointestinal symptoms. All of the gastrointestinal reactions that occurred during OFCs, whether individually or as part of a multisystem reaction, involved abdominal pain and vomiting. Of the 13 who initially presented with multisystem reactions, 10 passed their OFC. Only two of the 13 (15.4 %) who had an initial multisystem reaction also had one during the OFC. Twenty percent (2/10) of those who had a multisystem reaction during the OFC had such a reaction on initial presentation, and another twenty percent (2/10) had no or rare egg exposure previously. The majority of those who had a multisystem reaction had an initial reaction involving cutaneous findings only.

Failures to EH egg OFCs had a significantly higher rate of multisystem reactions than egg OFCs (100 vs 27 %; p = 0.01; Table 2). All of the EH egg failures involved multisystem reactions, and all occurred during the muffin stage. Introduction of EH egg products at home, in those with a history of only cutaneous reactions, and without asthma, has been suggested [8]. However, all of the EH egg OFC failures in this study resulted in multisystem reactions, and one occurred in a patient without asthma or severe egg allergy.

There are some study limitations. OFCs were not blinded, a common practice in the allergy community. With nearly 8 % of egg OFC failures involving only subjective complaints, blinded food challenges may have allowed for better interpretation of reactions. In addition, the goal dose of 12 g of egg protein may have contributed to a higher rate of GI reactions, as that may have been too much food for some children. We also followed conservative practices in the criteria for offering OFCs. Yet, the failure rate was nearly 22 %. Furthermore, history of initial reaction was based on parent recall, which may have over- or under- estimated the severity of the initial reaction, as well as the amount of egg involved in the initial reaction. Severe reactions during EH egg OFCs, in those with a history of mild reaction to egg, have been reported [3, 9]. Of those who presented with an initial multisystem reaction, the majority (8/13) did not have atopic dermatitis, a factor that contributes to the persistence of egg allergy [4]. Thus, these patients may have been more likely to resolve their egg allergy, explaining the lack of, or milder, reactions during the OFC.

This study showed that multisystem reactions during egg OFCs were not associated with initial multisystem reactions, and vice versa. Therefore, egg and EH egg OFCs should be done under medical supervision.
